# Sampling flying bats with thermal and near-infrared imaging and ultrasound recording: hardware and workflow for bat point counts

**DOI:** 10.12688/f1000research.51195.1

**Published:** 2021-03-08

**Authors:** Kevin Darras, Ellena Yusti, Andreas Knorr, Joe Chun-Chia Huang, Agus Priyono Kartono, Ilham .

**Affiliations:** 1 Agroecology, University of Göttingen, Göttingen, Niedersachsen, 37077, Germany; 2 CRC 990 - EFForTS, University of Jambi, Jambi, 36361, Indonesia; 3 Mess-, Steuerungs-, und Regeltechnik, University of Göttingen, Göttingen, Niedersachsen, 37077, Germany; 4 Division of Botanical Gardens, Taiwan Forestry Research Institute, Taipei, 10079, Taiwan; 5 Department of Forest Resources Conservation and Ecotourism, IPB University, Bogor, 16680, Indonesia

**Keywords:** bat, point count, thermal imaging, near-infrared, ecoacoustics, night vision, ecology

## Abstract

Bat communities can usually only be comprehensively monitored by combining ultrasound recording and trapping techniques. Here, we propose bat point counts, a novel, single method to sample all flying bats. We designed a sampling rig that combines a thermal scope to detect flying bats and their flight patterns, an ultrasound recorder to identify echolocating bat calls, and a near-infrared camera and LED illuminator to photograph bat morphology. We evaluated the usefulness of the flight pattern information, echolocation call recordings, and near-infrared photographs produced by our sampling rig to determine a workflow to process these heterogenous data types. We present a conservative workflow to enable taxonomic discrimination and identification of bat detections. Our sampling rig and workflow allowed us to detect both echolocating and non-echolocating bats and we could assign 84% of the detections to a guild. Subsequent identification can be carried out with established methods such as identification keys and call libraries, based on the visible morphological features and echolocation calls. Currently, a higher near-infrared picture quality is required to resolve more detailed diagnostic morphology, but there is considerable potential to extract more information with higher-intensity illumination. This is the first proof-of-concept for bat point counts, a method that can passively sample all flying bats in their natural environment.

## Introduction

Bats are nocturnal, flying mammals found in all continents except at the poles, and they perform a wide range of ecosystem functions ( Kunz *et al*., 2011). Ecologists, conservationists, and practitioners monitor the state of their populations to devise management plans, produce environmental assessments, or conduct basic research. However, detecting, counting, and identifying bats in their natural environment is challenging, because many bat species are not easily distinguishable morphologically ( Mayer & von Helversen, 2001) or acoustically ( Russo *et al*., 2017). Furthermore, their ultrasonic calls - which are also identification features - are mostly outside the human hearing range. To sample active bat communities effectively, it is currently advised to combine capture methods with ultrasound recording ( Flaquer *et al*., 2007; O’Farrell & Gannon, 1999). Both methods have limiting drawbacks but are required to identify species that have similar calls, those that have similar morphology, and especially those tropical bat species that do not emit echolocation calls at all (e.g., the vast majority of Pteropodidae). As a result, a comprehensive sampling of flying bats requires considerable resources.

Modern electronic components extend our sensing capabilities into new domains that can be useful for monitoring bats by providing different data types. For instance, the near- and mid-infrared electromagnetic spectrum ( International Organisation for Standardization, 2007) is typically used for surveillance applications and can aid the detection and identification of bats. Thermal imaging (i.e., mid-infrared, 3–50 µm wavelength) devices such as thermal scopes are increasingly adopted by rangers and hunters to detect wildlife at night and have recently become more available due to mass production. Thermal scopes detect temperature difference and can thus quickly detect warmer mammals - which are homeotherms - as has been done in caves for bats ( Betke *et al*., 2008). In contrast, the near-infrared light (0.78–3 µm) sensed by imaging sensors in night vision devices allows users to accurately capture detail even when no ambient light is available. Near-infrared illuminators can reveal animals using light that is invisible to most. Near-infrared imaging has been available for decades and can be used to survey and identify wildlife ( Blackwell *et al*., 2006), even over long ranges. Finally, ultrasound recorders for bioacoustics have become very affordable and dependable in field conditions. They are commonly used for monitoring and identifying bats over large areas, indiscriminately from all directions, by recording their ultrasonic echolocation calls ( Britzke *et al*., 2013). It follows that combining these different sensor types can theoretically enable thermal detection of bats, as well as identification of bats with complementary near-infrared and ultrasound information.

We developed and evaluated a novel method, called bat point count, to detect and identify flying bats at night. This method is analogous to bird point counts, where an observer stands in one point to listen to and sight flying vertebrates (i.e., birds), usually during the day. We integrated different devices to see in the dark and hear beyond the audible sound range, using thermal imaging for detection of bats, as well as near-infrared imaging and ultrasound recording for their identification. We derived detection statistics to find a workflow that effectively combines these different data types to process the majority of bat detections. We present that resulting workflow to enable taxonomic discrimination and identification of bats to the lowest-possible taxon level. Combining our sampling rig with our workflow yields the first proof-of-concept for a method that can passively sample flying bats, whether they are echolocating or not.

## Methods

### Study site and design

To showcase the utility of our sampling method, we sampled a bat community comprised of echolocating and non-echolocating bat species (i.e., Pteropodidae) in a tropical ecosystem, in the lowlands of Jambi province, Sumatra, Indonesia. We surveyed bats in May 2019 in three sites in an oil palm plantation inside the PT. Humusindo Makmur Sejati company estate (01.95° S and 103.25° E, 47 ± 11 m a.s.l.), near Bungku village. Our research project (EFForTS – CRC990) has a Memorandum of Understanding with the company to allow research activities. Oil palm plantations are relatively open below the canopy and afford unobstructed lines of sight to test our sampling method. The center of each sampling site was within 10 m of a river and 10 m of an unpaved road. One field team (EY and co-author I) sampled the sites on consecutive nights and repeated this twice so that each site was sampled three times. Each night, we conducted three 10-minute bat point counts in the first hour after nautical twilight, and three 10-minute bat point counts in the second hour. Each of the three point counts was directed either towards the road, river, or oil palm plantation.

### Hardware

**Figure 1.**
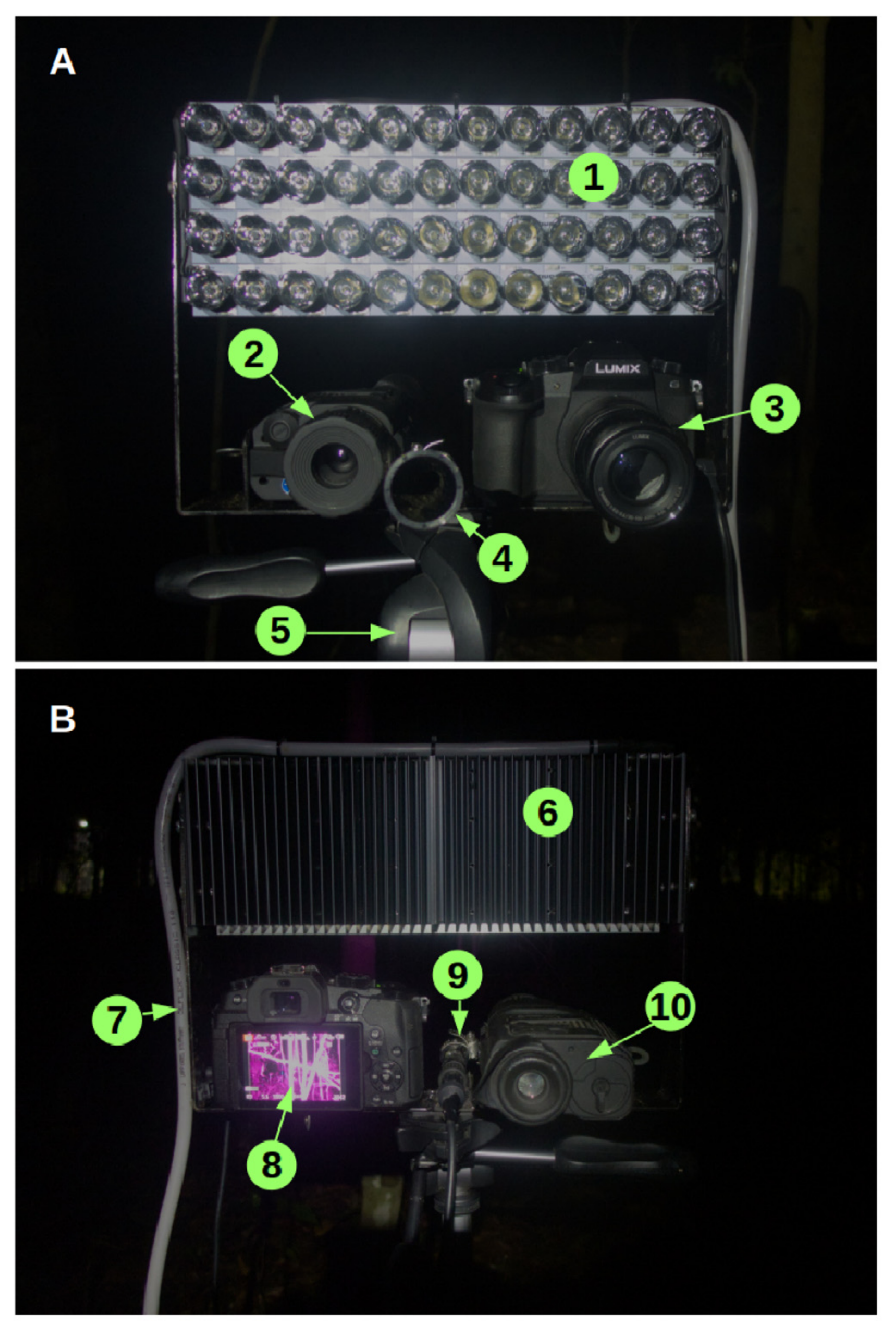
Sampling rig for bat point counts. View from the front ( **A**) and view from the observer or back ( **B**). 1: infrared illuminator (LEDs off, fitted with secondary optics); 2: thermal scope (front); 3: infrared camera (front); 4: ultrasonic microphone horn; 5: tripod head; 6: lamp heat sink; 7: cable for supplying power to the lamp; 8: infrared camera (back, with infrared live view); 10: thermal scope (back).

***Support rig*.** We constructed a sampling rig ( Figure 1, Table 1) for mounting three different devices sensing ultrasound, thermal, and near-infrared waves. We used a ball head tripod to mount a custom-built U-shaped aluminum slat designed to attach all devices safely and point them exactly in the same direction. Threaded holes allowed to attach the infrared and thermal imaging devices, which have the most common tripod socket type (1/4" diameter, 20 threads per inch) on their main bodies. At the top ends of the slat, the infrared illuminator was attached with screws through their radiators. The ball head tripod enables smooth and rapid movement for the observer to actively track flying bats. We connected a cabled remote shutter to the camera so that the tripod grip could be held in one hand and the shutter triggered with the other.

Our current mid- and near-infrared imaging setup, detailed below, was decided after trial-and-error with different devices. We tested digital rangefinders (Luna Optics) and optical night vision devices (Pulsar Challenger G2 3.5 × 56), infrared LED flashlights, small LED arrays fitted with secondary optics, and custom-adapted infrared digital cameras (Panasonic Lumix LX100). The ability to record high resolution infrared images and high thermal screen refresh rates were crucial.

***Thermal imaging*.** We use a thermal scope used for hunting (Quantum XQ19, Pulsar) to detect flying bats. That thermal scope has an upscaled VGA screen resolution of 640 × 480 pixels, from its microbolometer’s 384 × 288 pixels resolution, which - from our experience - is the lower usable limit for detecting small bats at a distance of several meters. It also has a refresh rate of 50 frames per second, allowing smooth tracking of fast-flying bats without delay. Its relatively broad field of view of 19.5 × 14.7 degrees (horizontal × vertical) is essential for observing a large area and being able to track rapidly-moving objects. We disabled the sleep function, the digital zoom, and the automatic calibration to avoid interrupting operation.

***Near-infrared imaging*.** We used a mirrorless interchangeable lens camera without its infrared filter for capturing near-infrared photographs. We removed the infrared filter ourselves ( Darras, 2018), but some companies provide that service for private customers. The camera (Panasonic Lumix G8) was coupled with a moderate zoom lens (Panasonic G Vario 35-100mm f/4.0-5.6). This camera was equipped with a fast memory card to shoot 10 frames per second, and it has a large buffer allowing users to shoot extended image bursts. A fast data pipeline is essential for capturing many successive steps of the rapid bat passes, which can last several minutes.

We zoomed the lens between 35 and 50 mm, depending on the distance of the bats. We set up the camera in manual mode with a shutter speed between 1/250 s (for slow-flying bats) until 1/500 s (fast-maneuvering bats), an aperture of F4 (at 35 mm) or F5.0 (at 50 mm). We adjusted the gain with the “Auto ISO” function with a maximum ISO of 6400. We used the continuous shooting mode with the “high” burst rate setting and electronic first-curtain shutter. We used the manual focus mode, auto white balance, and saved pictures as a JPEG in “high quality”, at full resolution, with a 4:3 aspect ratio. JPEG saturation, contrast, sharpening and noise reduction were all at minimum. The exposure metering mode was set to center, the screen brightness to minimum, sensor stabilization and autofocus assist lamp were off.

We maximise the photograph exposure with artificial lights. We attached a custom-built near-infrared lamp made of four 12-LED rigid, aluminium infrared LED strips. The LEDs were outfitted with secondary optics - lenses that focused the emitted infrared beam to a more narrow cone of 14 degrees FWHM (Full Width at Half Maximum: the angular width of the beam when the intensity at the edge is half the intensity in the center of the beam). The LED strips are cooled with vertically arranged radiators attached with screws into the lens attachment holes of the LED strips. Our setup required us to use plastic rings below the screws to avoid shorting the electrical circuit. We did not use visible light or visible light flashes to illuminate bats as to avoid disturbing them, and also to avoid attracting insect prey.

The near-infrared illuminator requires a custom continuous current supply unit built by AK ( Darras, 2021). The power source is a lithium-polymer 4500 mAh (66 Wh) battery, four voltage converters are each powering one LED strip with a constant 0.7 A. The power adapter is turned on with a toggle switch on the side of the toolbox; a green LED indicates when it is powered. The battery capacity theoretically lasts for 30 min of use and there is currently no indication of the battery charge. To avoid over-discharge, the battery should be recharged after 20 minutes, using a balanced charger.

***Ultrasound recording*.** We used an open-design full-spectrum microphone, capable of recording audible sound or vocal notes, as well as ultrasound bat calls (Parus microphone, ( Darras *et al*., 2018)). We attached the microphone to the sampling rig with rubber bands to isolate it from handling noise; it was fitted with an ultrasound horn (Wildlife acoustics) to make the microphone more directional and sensitive to the sound coming from the direction we pointed it to. The ultrasonic horn, even though it makes more directional recordings, does not entirely exclude echolocation calls from outside of the thermal scope’s field of view, especially when bats are close to the microphone. The microphone was connected to an acoustic recorder (SM2Bat+, Wildlife Acoustics) with a 3 or 5 m cable, laid on the ground below the tripod. It recorded mono audio (on one channel) at a 384 kHz sampling frequency in WAV format, without triggers.

### Workflow

***Field procedure*.** At each sampling site, we determined the thermal detection area by measuring the maximum detection distance in the three observation directions (oil palm, river, and road), each separated by 120 degrees. We pointed the thermal scope at the hand of co-author I facing away from the thermal scope to gauge at what maximal distance bats, that are roughly as large as a human hand, are still visible in the thermal scope. We then measured the distance of co-author I using a rangefinder (pointed at a hand-held whiteboard (A4 sheet dimensions) acting as a reflector, illuminated with a headlamp to facilitate aiming at it.

Before sampling each direction, we observed in the infrared camera where the bats’ flyways generally are. We then held the whiteboard at the most often used distance in flyways, measured its distance with the laser rangefinder, and wrote it on the whiteboard along with the site code and the direction (oil palm, road, river). The whiteboard was used to autofocus the infrared camera to that distance to clearly identify the site. We used a dedicated smartphone app to calculate the depth of field at the corresponding focal length, subject distance, and aperture (given the sensor size), making sure it was at least four meters wide.

We set up devices on the sampling rig and turned them all on, aligning the direction of the thermal scope and infrared camera precisely. We checked that the camera and ultrasound recorder times were synchronous. We started manual recordings and mentioned the name of the plot and direction vocally. Ten minute counts were measured with a timer. We used the thermal scope to scan the field of view (120 decimal degrees) in front of the observer (EY) up and down, then shifted the frame sideways, systematically, until a flying bat was detected. When a bat was detected, the observer ordered the assistant to switch the LED illuminator on, mentioned "bat detected", and took infrared pictures. The bat was described while tracking it, recording the flight pattern (“insectivorous” when maneuvering, “frugivorous” when not maneuvering), activity (flying, hunting, hanging, etc.), and the number of individuals. When the bat was gone, the observer said "bat gone". The observer rested for a maximum of 5 minutes until the next point count.

***Processing ultrasound recordings*.** Ultrasound recordings were retrieved from the recorder and uploaded in BioSounds ( Darras *et al*., 2020) to annotate the thermal and non-thermal bat detections. Non-thermal detections are bat passes that were picked up only by the ultrasonic microphone - they were not detected by the observer because the bat was outside of the thermal scope’s field of view. We zoomed into the recording and scrolled through it until bat calls or camera shutter sounds were found to determine the bat detection timings. The start and end timings of a thermal detection were determined from the vocal mentions (“bat detected/gone”). The start and end timings of an ultrasound-only detection were determined from the start and end of the bat pass based on the visible echolocation calls; we defined bat passes as sequences of at least two calls less than one second apart. Ultrasound recordings were screened by EY at a magnification of 860 pixels/60 s and cross-checked by co-author I. We noted the number of individuals mentioned by the observer in the annotation for thermal detections. Bat passes from the same individual that were separated by less than 10 seconds were merged into one tag. For thermal detections, we entered an ID into the annotation, thereby linking it to a table containing their vocally mentioned observation data. All detected bats were flying.

Each annotation with echolocation calls was directly assigned a bat call type or species. We compared bat calls against our own reference collection of bat calls obtained from captured bats (Chiroptera reference collection in BioSounds) to identify the calls to species; when inconclusive calls were found, we assigned them to call types. Within each recording, we measured call parameters for each bat species and call type that was recorded clearly (to avoid biased call parameters from distant calls). We measured call parameters based on the three strongest, not saturated calls: call duration (duration of a single pulse), inter-call interval (time from the start of one call to the onset of the next), start frequency (frequency value at the start of the call), end frequency (frequency value at the end of the call) and peak frequency (frequency with maximum energy for the whole call).

**Figure 2.**
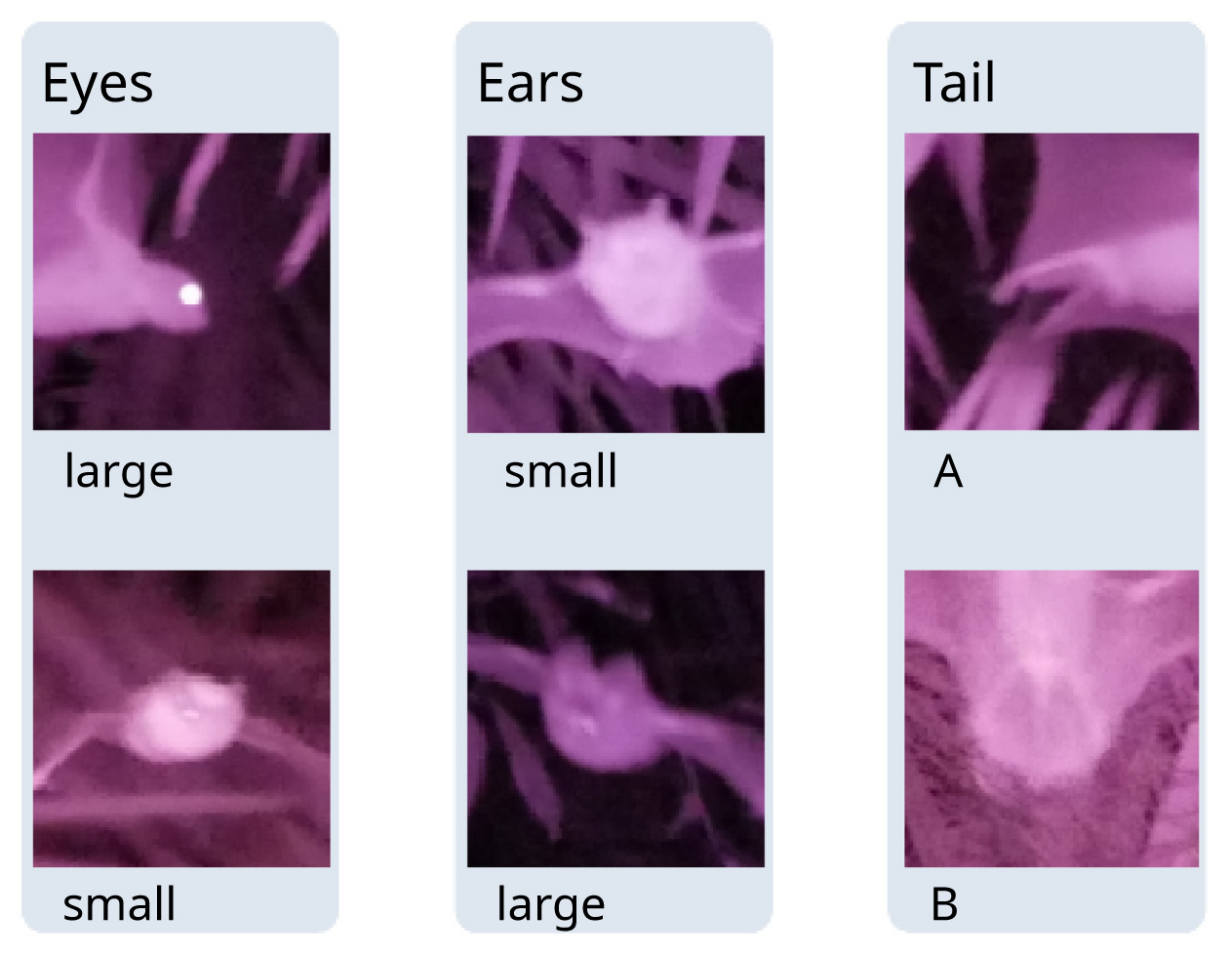
Near-infrared photographs showing diagnostic morphological features, obtained with bat point counts. Pictures were cropped from the original.

***Processing infrared images*.** We used infrared imagery to confirm whether detections are from insectivorous (echolocating) or frugivorous (non-echolocating) bats using visible morphological features. Using the photograph meta-data (Exif), the infrared images were automatically assigned to the corresponding detections based on their timings (see R script in ( Darras, 2021). Images with ambiguous timings were manually assigned to detections. For each point count, we counted the number of images shot and the number of images containing a bat image to compute the hit rate. For each detection, we noted the number of images containing a bat. We used the latter to record the presence of diagnostic morphological features ( Figure 2) into our observation table: we distinguished between small eyes (characteristic of insectivorous bats) and large eyes (characteristic of Pteropodidae); we distinguished large ears (only found in insectivorous bats such as Hipposideridae, Rhinolophidae, Megadermatidae, Nycteridae) from smaller ears (found in Pteropodidae and insectivorous bat families as well), we distinguished between tail types (A: short tail, not enclosed in reduced interfemoral membrane, as in Pteropodidae, B: tail enclosed in interfemoral membrane as in Rhinolophidae, Hipposideridae, Vespertilionidae, Miniopteridae ( Srinivasulu *et al*., 2010)).

***Determining the workflow*.** We assessed the usefulness of the different data types obtained with bat point counts: the observed flight pattern, the near-infrared bat photographs, and the ultrasound recordings. We used infrared imagery to generate subsets of confirmed insectivorous (having large ears, B-type tails or small eyes) and frugivorous (having large eyes or A-type tails bats). Among those, we counted for how many detections each flight pattern type could be observed and the number of detections with and without echolocation calls. We computed the total hit rate (proportion of photos with bats) and the useful hit rate (proportion of photos with bats that had a visible aspect of the morphology). Based on our results, we prioritised the different data types and devised a workflow for taxonomic discrimination and identification to the lowest possible level.

***Software and hardware***

**Table 1.** List of components used for building a sampling rig and carrying out bat point counts.

Component	Model	Source
Software		
Analysis	R 3.6.3	R core team - https:// cran.r-project.org/bin/
Ecoacoustics	BioSounds	www.biosounds.uni- goettingen.de/
Depth of Field calculator	PhotoPills DOF calculator (not free anymore but online version is)	PhotoPills - www. photopills.com/ calculators/dof
Near infrared illuminator (custom assembly)		
12-LED array	4 V3 Infrared PowerBars	www.leds.de
Array-to- power supply connector	9-pin D-subminiature plug	electronics store
LED	Oslon 4715SH LEDs (soldered on array)	Osram
Secondary optic	Tina2-RS	LEDiL – www.ledil. com/
heat sink		electronics store
Power supply unit (custom assembly)		
Printed circuit board	design files provided in extended data	Custom build
Battery	Lithium-polymer 4500 mAh 14.8 V, 25C	Red Power - www. reichelt.com/
Voltage converter	4 LDH-45A-700 (soldered on printed circuit board)	MeanWell - www. reichelt.com/
Toolbox	For housing the power supply unit	Krisbow (Indonesia)
Battery- to-charger connector	Type: LP4	Molex
Balanced charger	Xpeak 50 BAL charger charging settings: LiPo BALANCE mode, 3.0A, 14.8V, 4S	Xpeak - www.reichelt. com/
Sensors		
Tripod	Compact Advanced (ball- head)	Manfrotto
Metal rig	design files provided in extended data	Custom build
Thermal scope	Quantum XQ19	Pulsar
Near- infrared camera	Lumix G8 (without infrared filter)	Panasonic
Camera lens	G Vario 35-100mm f/4.0- 5.6	Panasonic
Memory card	Extreme Pro 64 GB, UHS3	Sandisk
Full- spectrum Microphone	Parus, open-source Sonitor microphone system	Custom build
Ultrasonic horn and Sound recorder	SMBat2+	Wildlife Acoustics - www.wildlifeacoustics. com/
Laser rangefinder	LASER 1000 AS	Nikon

## Results

We obtained 109 thermal bat detections, shot 3152 near-infrared photos, and recorded 9 hours of ultrasound. Out of three recording hours per site, 56 minutes were occupied by ultrasound bat passes (31 % on average). The thermal detection ranges averaged 48 m with no noticeable differences between directions; 15 of the thermal detections were only detected thermally. There were 810 ultrasound-only detections. Out of the 109 thermal detections, photos with bats were found in 60 detections (55 %), and flight maneuvers were noted in 50 detections (46 %). Out of the detections with bat photos, 45 had a visible aspect of the bat morphology (41 % of thermal detections). Out of the 3152 near-infrared photos, 981 (3 to 46 % per night, with an average of 25 %) contained bats; 154 were too dark because the LED illuminator was not turned on in time or the battery was too low. A bat pass video feed from a maneuvering bat seen in the thermal scope is available as extended data ( Darras, 2021).

### Workflow

Confirmed frugivorous bat detections often had no simultaneous echolocation calls (85 %) and non-maneuvering flight patterns (both 71 %). We found seven confirmed frugivorous bat detections with visible large eyes, one had a visible tail characteristic of Pteropodidae. One of these had simultaneous echolocation calls, and two had a maneuvering flight pattern. Large reflective eyes were also visible from far away, even before the bat body was clearly visible, and also when the bat was flying sideways to the observer ( Darras, 2021). Frugivores’ tails were generally not observed as they were generally gliding low and not maneuvering much.

Confirmed insectivorous bat detections almost always had simultaneous ultrasound (97 %) and often had maneuvering flight patterns (78 %). We found 31 confirmed insectivorous bat detections with either small eyes (31), large ears (1), or a visible tail with a patellum (with the tail enclosed in the interfemoral membrane) (33). Only one of these detections had no simultaneous echolocation call, and eight had a non-visible flight pattern.

**Figure 3.**
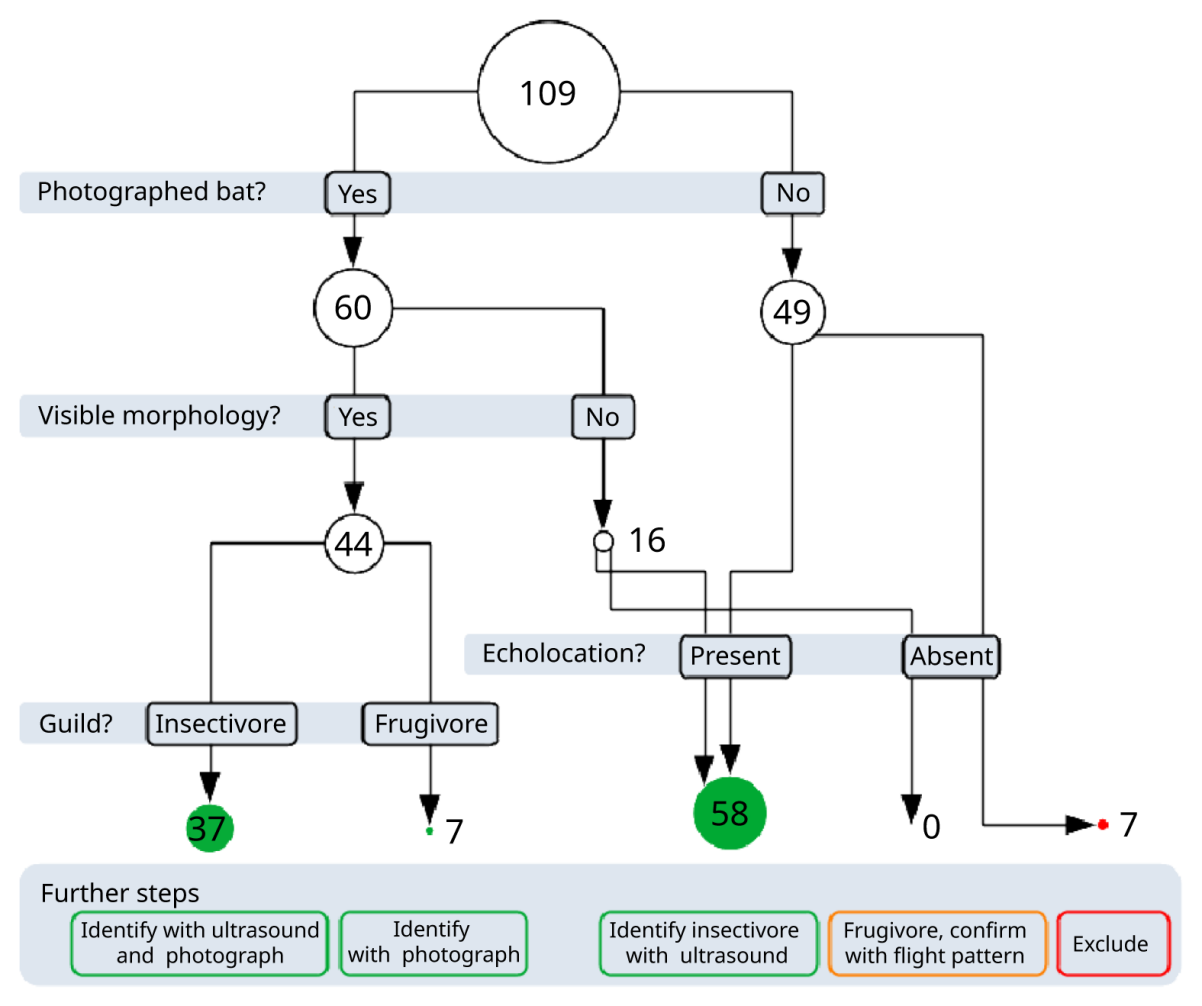
Workflow used for discriminating bat taxa with bat point count data. The circle diameter scales with the number of detections. Green disks correspond to detections where species identification can theoretically be achieved; orange corresponds to detections where identification could be achieved on the family level; red corresponds to those where no identification is possible.

Based on these results, we devised the following workflow for assigning taxonomic identifications of thermal bat detections ( Figure 3). When detections had infrared photographs showing clearly visible aspects of the bat morphology, they were used to determine the guild; insectivores were identified using the simultaneously recorded echolocation calls and infrared imagery was used to confirm and narrow down their identity; frugivores were identified based on the infrared imagery. We excluded detections without any near-infrared photographs or ultrasound. For detections that had bat photos but no visible bat morphology, we considered the bat to be inside our detection range for ultrasound: When there were simultaneous echolocation calls, we deemed the bat to be insectivorous and used the calls to determine its identity; When there were no simultaneous echolocation calls, we deemed the bat to be frugivorous but could not identify it further than the family level. Using this workflow, 84% of bat detections could be assigned to a guild and subsequently identified using ultrasound and near-infrared data.

## Discussion

For the first time, we show that it is possible to combine thermal, ultrasonic, and infrared sensors to comprehensively sample both echolocating and non-echolocating bats flying at night with bat point counts, and we propose a conservative workflow for taxonomically discriminating or identifying most bat detections. Subsequent identification can be carried out using established morphological keys and acoustic call libraries.

Echolocation calls contain rich taxonomic information for identifying echolocating bats, usually up to species- or genus-level, and at least to the family level. Currently, the most direct information source for detecting and identifying echolocating bats from bat point counts is ultrasound. However, we need to ensure that the recorded calls come from the currently detected bat. It is difficult to constrain the pickup angle of ultrasound (compared to light), even when using microphone attachments such as our horn. As a result, during any detection, it is unclear whether simultaneously recorded echolocation calls come from the bat visible in the thermal scope or surrounding echolocating bats. However, even though it is possible that we are identifying another bat in the surroundings, we would still identify a nearby echolocating bat which belongs to the community of the sampling site that would likely be detected at another time point. As a result, such an identification mistake does not constitute a very problematic false positive. Luckily, we could confirm that usually, simultaneously recorded ultrasound calls should come from the bat spotted in the thermal scope.

Additionally to ultrasound, bat point counts generate observational data that are complementary or unique. First, the observed flight pattern is potentially useful for determining a bat’s guild and narrowing down the identification candidates. However, we found that it could not be discerned with certainty for most of the detections due to the short duration of detections of bats that are just quickly passing through the sampling site. We could still use this information to confirm the guild and rough taxonomy (Pteropodidae vs. non-Pteropodidae) of the detected bat. Second, near-infrared photographs allowed us to detect bats that are inherently not detectable with ultrasound. In many instances, it was possible to use the size of the eyes as a clear detection proof for Pteropodidae. However, the picture quality was only sufficient for determining some aspects of the bat morphology, such as the eyes, ears, and shape of the tail. These rough indications are generally not sufficient for species-level identification, which relies on accurate measurement of body parts. Thus, we cannot identify non-echolocating bats yet with the same precision as echolocating bats, which additionally provide ultrasonic cues for detection. However, we are currently able to detect all bat types reliably for a comprehensive assessment of flying bat communities, which represents a significant contribution to bat sampling methods.

Capturing flying bats at night is the next photographic frontier, because it is technically extremely challenging. *Ambient light levels are very low*: artificial illumination with infrared light and capture using modified cameras is necessary. *Bats fly fast:* we determined that minimal shutter speeds of 1/250 s are needed for “freezing” the movement of relatively slow-flying frugivorous bats, and 1/500 s for insectivores. *Bats are small:* narrow fields of view (a large "zoom" value or focal length) are needed to make them fill the photographic frame so that we have enough resolution to identify them. We tried to strike the best balance between depth of field, focal length (zoom), and shutter speed. As a result, even with our 48 near-infrared LED illuminator, many of our images are unclear: unsharp because they are outside the relatively shallow depth of field, with too low resolutions because they are too far away, and with significant image noise because of insufficient illumination.

A meticulous balance between the opposed technical requirements is needed for obtaining high-quality near-infrared imagery. The narrower the field of view, the shallower the depth of field - the range of distances, in front and to the back of the focal plane, with acceptable sharpness. The angle of view also cannot be too narrow, as we need to be able to rapidly track bats to capture them inside the photographic frame - roughly one quarter of our photos contained bats. The depth of field also needs to be large as we cannot predict the highly variable distances of the bats, and it is currently impossible to autofocus on such small and fast subjects. We can enlarge the depth of field to some degree by using small apertures, but they considerably decrease the light that reaches the sensor. It follows that the usefulness of bat point counts is currently primarily limited by the near-infrared illumination. This limitation may be overcome with stronger near-infrared lighting to increase the proportion of usable photographs and the depth of identification for non-echolocating bats, and for echolocating bats that are not easily determined through their calls.

We identified several other technical challenges and areas of improvement, which the scientific community can work on to continue enhancing bat point counts. The picture quality is also limited by the resolution of the lens and sensor combination. This limitation may be overcome with sharper lenses, higher-resolution cameras shooting higher continuous photo burst rates to increase the chances of obtaining usable pictures. Thermal imaging with higher resolutions and higher frame rates would probably only bring modest improvements to the tracking ease, determination of flight pattern, and hit rate. Human operation is currently still needed to actively track bats; developing an automated tracking system is challenging and implies to integrate thermal and infrared sensors into a common design. We currently use horns for focusing sound waves, but should consider parabolic reflectors to have a more narrow sound pickup pattern. Possibly, greater directivity could also simply be attained with directional microphones, but this remains to be tested. We welcome prospective co-authors to contribute to our work and update the methodological study of bat point counts with new findings.

## Data availability

### Underlying data

Open Science Framework: Bat point counts - design, analysis, images & data

https://doi.org/10.17605/OSF.IO/ZNVRY ( Darras, 2021)

This project contains the following underlying data:

- Plots.csv – plot-level data
- Surveys.csv – survey-level data
- BioSounds tags.csv – annotation data exported from BioSounds
- analysis – technical.R – R script for reproducing analysis

### Extended data

Open Science Framework: Bat point counts - design, analysis, images & data

https://doi.org/10.17605/OSF.IO/ZNVRY ( Darras, 2021)

This project contains the following extended data:

- photos move.R – R script for assigning images to detections
- bat pass thermal scope.mp4 – video of a flying bat
- detection 262 – diving.gif – diving maneuver of a bat
- detection 114 - H orbiculus.gif – *Hipposideros orbiculus* with large ears
- detection 256 - reflective eyes.gif – Pteropodid with large eyes
- ao004.T3001 – TARGET file for the printed circuit board layout
- ao004_SCH.PDF – PDF showing the printed circuit layout

Data are available under the terms of the Creative Commons Zero "No rights reserved" data waiver (CC0 1.0 Public domain dedication).
