# Supplementary material for: Sampling flying bats with thermal and near-infrared imaging and ultrasound recording: hardware and workflow for bat point counts
Source: F1000Res. 2022 Mar 23;10:189. Originally published 2021 Mar 8. [Version 2] doi: 10.12688/f1000research.51195.2 (PMC8987345; doi:10.12688/f1000research.51195.2)
Supplement: Peer review history

# Peer review: Reviewer response for version 2

**DOI:** 10.5256/f1000research.122547.r128591

**Published:** 2022-04-06

**Authors:** Sumit Dookia

**Affiliations:** 1 University School of Environment Management, Guru Gobind Singh Indraprastha University, Dwarka, New Delhi, 110078, India

I have seen the entire document and approve the paper in this stage.

Is the rationale for developing the new method (or application) clearly explained?

Yes

Is the description of the method technically sound?

Yes

Are the conclusions about the method and its performance adequately supported by the findings presented in the article?

Yes

If any results are presented, are all the source data underlying the results available to ensure full reproducibility?

Yes

Are sufficient details provided to allow replication of the method development and its use by others?

Yes

Reviewer Expertise:

NA

I confirm that I have read this submission and believe that I have an appropriate level of expertise to confirm that it is of an acceptable scientific standard.

# Peer review: Reviewer response for version 1

**DOI:** 10.5256/f1000research.54331.r101541

**Published:** 2022-02-07

**Authors:** Sumit Dookia

**Affiliations:** 1 University School of Environment Management, Guru Gobind Singh Indraprastha University, Dwarka, New Delhi, 110078, India

Authors tried to combine different devices to let them work in unison for detecting one of the most fascinating as well as challenging mammals, which flies in complete darkness. No critical comments on the manuscript, well defined methods and also a good degree of acceptance of the limitation of outcome in the form of bat detection viz a viz photographs and ultrasonic recording. This experiment needs repetition at different places to validate the accuracy of the results. The present setup is enough for bat monitoring (up to family level with little more refinement) purpose. 

**Minor Comment/Suggestion: **

- A better and clear picture of the experimental setup is required. 
- In Fig. 1, Sl. No. 9 is not defined or mentioned. Need to be defined.   
- Do authors have a checklist of total bat species present in that study area, if yes, then what was the detection probability through this exercise and how efficiently it is detecting?

Overall, it is a very interesting study and needs many replications at different locations.    

Is the rationale for developing the new method (or application) clearly explained?

Yes

Is the description of the method technically sound?

Yes

Are the conclusions about the method and its performance adequately supported by the findings presented in the article?

Yes

If any results are presented, are all the source data underlying the results available to ensure full reproducibility?

Yes

Are sufficient details provided to allow replication of the method development and its use by others?

Yes

Reviewer Expertise:

NA

I confirm that I have read this submission and believe that I have an appropriate level of expertise to confirm that it is of an acceptable scientific standard, however I have significant reservations, as outlined above.

# Response

**Published:** 2022-03-16

**Authors:** Kevin Darras

**Affiliations:** University of Goettingen, Germany

Thank you very much for your assessment of our study and constructive comments.

 We provide a clearer picture of the experimental setup and fixed the numbering of the parts. Figure 1 has been updated with daylight photographs of the setup, as well as two additional panes showing the setup and the live view at night.

 We provide a checklist of the species detected by different sources and methods in the extended data now. We also added this to the discussion to acknowledge and address your comment about validation and detectability, referring to our corresponding, in-depth ecological study that was recently published.

# Peer review: Reviewer response for version 1

**DOI:** 10.5256/f1000research.54331.r84435

**Published:** 2021-05-17

**Authors:** Christian Vincenot

**Affiliations:** 1 Department of Social Informatics, Kyoto University, Kyoto, Japan

Very interesting work. I have no criticism to advance, as the work is methodologically sound and the manuscript well written (esp. the discussion section, which already describes well the limitations of the approach and future system developments). I just hope to see this line of work developed further and tested on more challenging cases (i.e. categorization between finer guilds or species). But this is clearly a promising preliminary study of a versatile tool that deserves to be indexed.

 Minor comments:

- Do the authors have a list of species present in their study area? It would be interesting to assess how challenging (and useful) the present assessment was, considering that Pteropodidae for instance come in various sizes, hence making the ID more or less difficult.
- Out of curiosity, I would have liked to see the breakdown of the results of the different discrimination steps used to analyse the IR images.
- Dimensions as well as a better image of the whole system would be nice. (Fig. 1 is good but seems to show only part of the device, does it not?) Furthermore, a picture showing the deployment settings would also be good.

Is the rationale for developing the new method (or application) clearly explained?

Yes

Is the description of the method technically sound?

Yes

Are the conclusions about the method and its performance adequately supported by the findings presented in the article?

Yes

If any results are presented, are all the source data underlying the results available to ensure full reproducibility?

Yes

Are sufficient details provided to allow replication of the method development and its use by others?

Yes

Reviewer Expertise:

Bat Ecology and Conservation, Computer Science.

I confirm that I have read this submission and believe that I have an appropriate level of expertise to confirm that it is of an acceptable scientific standard.

# Response

**Published:** 2022-03-16

**Authors:** Kevin Darras

**Affiliations:** University of Goettingen, Germany

Thank you very much for your speedy assessment of our study and constructive comments. We now received two reviews and revised it; sorry for the lateness.

 We provide a checklist of the species detected by different sources and methods in the extended data now and elaborate on it in the discussion’s first paragraph, referring to our corresponding in-depth ecological study that was recently published.

 We included some photographs illustrating the discrimination steps of the IR image analysis into Figure 3.

 We provide a clearer picture of the experimental setup. Figure 1 has been updated with daylight photographs of the setup, as well as two additional panes showing the setup and the live view at night.
